# Evaluation of the efficacy of retroperitoneoscopic debridement for lumbar tuberculosis: a retrospective study and preliminary results

**DOI:** 10.1007/s00264-026-06803-5

**Published:** 2026-04-23

**Authors:** Yongrui Yang, Wenkai Ruan, Jianlong Li, Rongpan Dang, Huigang An, Wentao Zhao, Yingxin Zhao, Liang Xu, Hongdong Tan

**Affiliations:** Department of Orthopedics, Shandong Public Health Clinical Center, Shandong University, Jinan, Shandong China

**Keywords:** Lumbar tuberculosis, Retroperitoneoscopic debridement, Endoscopic technology, Minimally invasive surgery

## Abstract

**Background:**

Lumbar tuberculosis (LTB) is a significant global health concern, often requiring surgical intervention when medical treatment is insufficient. Retroperitoneoscopic debridement offers a minimally invasive approach to manage LTB, potentially reducing complications and recovery time compared to traditional open surgery. However, its efficacy and safety remain understudied. This retrospective cohort study aims to evaluate the clinical outcomes, complication rates, and long-term effectiveness of retroperitoneoscopic debridement in patients with LTB.

**Methods:**

This retrospective cohort study analyzed patients with LTB and treated with retroperitoneoscopic debridement at our institution from July 2022 to July 2023. Baseline patient characteristics, operative time, operative blood loss, changes in inflammatory markers (e.g., CRP, ESR), complication rates, the visual analog scale (VAS) scores of the back, Oswestry Disability Index (ODI) scores, kyphotic angle changes in infective level and radiological follow-up outcomes were recorded.

**Results:**

Twenty patients with LTB were finally included. The mean operative time, operative blood loss, and postoperative drainage volume were 88.42 ± 7.07 min, 26.32 ± 10.61 ml, and 58.00 ± 11.31 ml, respectively. The mean follow-up time was 20.21 ± 1.41 months. During the follow-up, both VAS score and ODI score were significantly improved at one month, three months postoperative, and the final follow-up, compared with preoperative (*P* < 0.001). At the final follow-up, the kyphotic angle in the infective level remained good in all patients and no spinal instability was observed. Bone graft fusion rate at the final follow-up was 100%. Compared with preoperative, ESR and CRP were both showed significant decrease at one and three months postoperative (*P* < 0.001). One patient was found with postoperative complications, and cured after active treatment.

**Conclusion:**

Retroperitoneoscopic debridement appears to be a safe and effective minimally invasive approach for treating LTB. However, long-term efficacy requires further validation through prospective studies with larger sample sizes and extended follow-up periods.

## Introduction

Lumbar tuberculosis (LTB), a form of spinal tuberculosis caused by Mycobacterium tuberculosis, remains a significant global health challenge, particularly in developing countries where TB prevalence is high [1]. It accounts for approximately 50% of skeletal TB cases and can lead to severe complications such as spinal deformity, neurological deficits, and chronic pain if not adequately managed [2, 3]. While anti-tuberculous chemotherapy is the cornerstone of treatment, surgical intervention is often required in cases with significant bony destruction, abscess formation, or neurological impairment [4]. Traditional open surgery, such as anterior or posterior debridement and fusion, has been effective but is associated with substantial tissue trauma, prolonged recovery times, and higher risks of complications [5, 6].

In recent years, minimally invasive techniques have gained attention for their potential to reduce surgical morbidity while achieving comparable clinical outcomes [7, 8]. Retroperitoneoscopic debridement, an endoscopic approach, allows for targeted removal of infected tissue and abscesses through small incisions, minimizing damage to surrounding structures [9]. This technique has shown promise in treating LTB by offering reduced blood loss, shorter operative times, and faster recovery compared to open surgery [10]. However, despite its increasing use, there is limited evidence on its long-term efficacy, safety profile, and impact on functional outcomes. This study aims to evaluate the clinical effectiveness of retroperitoneoscopic debridement in patients with LTB, focusing on pain relief, functional improvement, and complication rates.

## Materials and methods

This study was approved by the ethics committee of Shandong Public Health Clinical Center, Shandong University (approval number K2025137). Written informed consent was obtained from all patients.

### Patient population

This study included a total of 20 patients with LTB who underwent retroperitoneoscopic debridement and bone graft fusion from July 2022 to July 2023. Among the 20 patients, 11 were male (11/20, 55%) and nine were female (9/20, 45%), with a mean age of 55.6 ± 19.09 years.

Inclusion Criteria: (1) Age greater than 18 years; (2) Diagnosis of lumbar tuberculosis (LTB) confirmed by computed tomography (CT)-guided biopsy or comprehensive analysis based on imaging findings, clinical symptoms, T-SPOT.TB test, and Xpert MTB/RIF assay; (3) Single-segment lumbar tuberculosis (L1/2–L4/5); (4) The operative method was retroperitoneoscopic debridement; (5) Follow-up duration of more than 12 months.

Exclusion Criteria: (1) Patients with incomplete clinical data or missing follow-up records; (2) Patients with contraindications to surgery or those who refuse surgical treatment; (3) Asymptomatic patients; (4) Patients with previous spinal surgery history.

All patients received standard anti-tuberculosis treatment. Additionally, data were collected on the following parameters: infective level, associated medical illness, T-SPOT, X-pert, pathology, antibiotics, preoperative and postoperative C-reactive protein (CRP) and erythrocyte sedimentation rate (ESR) levels at 1 and 3 months, preoperative and postoperative Visual Analog Scale (VAS) scores, Oswestry Disability Index (ODI) scores, changes in kyphotic angle at the infective level, operative time, operative blood loss, postoperative drainage volume, timing of drain removal, outcomes assessed by the modified MacNab criteria (MNC), fusion status, and postoperative complications. Detailed information is presented in Tables 1, 2, 3, 4.

**Table 1:** Clinical and treatment characteristics

Case	Age	Sex	Level	Associated medical illness	T-SPOT	X-pert	pathology	Antibiotics
1	72	F	L3/4	Bronchiectasis	positive	positive	MT	HRZE + V
2	63	F	L3/4	HTN	positive	positive	MT	HRZE + V
3	39	M	L1/2	NONE	positive	positive	MT	HRZE + V
4	39	F	L3/4	NONE	positive	positive	MT	HRZE + V
5	55	M	L2/3	HTN/DM	negative	positive	MT	HRZE + V
6	73	M	L2/3	HTN	positive	positive	MT	HRZE + V + Lzd
7	62	F	L3/4	NONE	positive	positive	MT	HRZE + V
8	46	F	L3/4	NONE	positive	positive	MT	HRZE + V
9	78	M	L2/3	DM/CHD	positive	positive	MT	HRZE + V
10	42	F	L4/5	NONE	positive	positive	MT	HRZE + V
11	58	M	L3/4	DM	positive	positive	MT	HRZE + V + Lzd
12	63	F	L1/2	NONE	positive	positive	MT	HRZE + V
13	56	M	L3/4	HTN/DM	positive	positive	MT	HRZE + V
14	49	M	L1/2	DM	negative	positive	MT	HRZE + V
15	67	F	L3/4	NONE	positive	positive	MT	HRZE + V
16	58	F	L2/3	PE	positive	positive	MT	HRZE + V + Lzd
17	42	M	L4/5	SLE	positive	negative	MT	HRZE + V
18	56	M	L3/4	DM	positive	positive	MT	HRZE + V
19	49	M	L1/2	NONE	positive	positive	MT	HRZE + V
20	45	M	L4/5	HTN/DM	positive	positive	MT	HRZE + V

Abbreviations: *HTN* Hypertension, *DM* Diabetes Mellitus, *CHD* Chronic Heart Disease, *PE* Pulmonary Embolism, *SLE* Systemic Lupus Erythematosus; *MT* Mycobacterium tuberculosis, *HRZE* Isoniazid Rifampicin Pyrazinamide Ethambutol; *V* levofloxacin, *Lzd* linezolid, *F* Female, *M* Male

**Table 2:** Inflammatory markers preoperative and postoperative

Number	CRP(mg/dL)			ESR(mm/h)		
	Pre	1M	3M	Pre	1M	3M
1	64.46	16.45	1.23	62	20	12
2	46.23	12.32	4.04	55	21	19
3	67.28	24.61	5.41	78	19	20
4	68.02	25.16	4.23	45	14	8
5	83.17	12.06	6.03	72	21	6
6	19.26	10.14	6.62	90	22	15
7	32.15	12.08	4.39	66	12	9
8	42.21	22.32	3.06	110	16	12
9	44.19	15.37	4.24	92	36	20
10	88.32	9.32	6.08	64	29	19
11	67.42	3.74	1.50	67	17	12
12	55.15	4.23	5.25	78	20	8
13	42.38	10.09	6.72	49	16	12
14	50.12	11.24	3.67	56	28	16
15	26.07	1.37	0.52	36	12	6
16	124.92	6.05	3.67	46	25	9
17	98.34	7.36	4.86	67	12	7
18	56.28	6.06	2.45	70	23	12
19	65.35	15.23	6.31	37	19	8
20	60.02	5.20	1.22	86	18	10

**Table 3:** Pre- and post-operative clinical outcomes

Parameter	Pre	1 Month	3 Months	Final follow-up
VAS	6.53 ± 1.48	4.37 ± 1.23	2.24 ± 0.96	1.13 ± 0.85
ODI	45.37 ± 4.24	32.71 ± 3.14	20.13 ± 2.68	9.28 ± 2.48
Kyphotic Angle	7.52 ± 4.19	8.25 ± 3.26	8.28 ± 4.13	9.91 ± 4.46

**Table 4:** Surgical characteristics

Case	Operative time(min)	Operative blood loss(ml)	Postoperative drainage volume(ml)	Drain removal timing(days)	MNC	Fusion	Complication	Follow-Up (months)
1	85	20	78	5	Excellent	Yes	None	24
2	80	15	46	6	Good	Yes	None	22
3	90	20	32	4	Good	Yes	None	18
4	105	30	90	5	Good	Yes	None	20
5	90	40	78	7	Excellent	Yes	None	18
6	95	20	78	4	Good	Yes	None	24
7	70	25	66	10	Good	Yes	None	18
8	75	45	78	8	Good	Yes	None	20
9	80	20	40	7	Good	Yes	None	24
10	95	25	30	6	Good	Yes	None	18
11	85	30	54	5	Good	Yes	None	18
12	105	30	48	5	Fair	Yes	None	20
13	90	25	68	6	Good	Yes	None	18
14	80	15	82	7	Good	Yes	None	18
15	95	20	70	5	Good	Yes	None	18
16	100	25	80	8	Good	Yes	None	24
17	70	30	48	6	Excellent	Yes	None	20
18	90	25	48	10	Fair	Yes	Transient	22
19	95	30	36	6	Good	Yes	None	24
20	90	30	30	7	Good	Yes	None	20

Abbreviations: *LTB* Lumbar tuberculosis, *CRP* C-Reactive Protein, *ESR* Erythrocyte Sedimentation Rate, *VAS* Visual Analog Scale, *ODI* Oswestry Disability Index, *MNC* MacNab Criteria, *AP* AnteroPosterior

### Surgical technique

1. Positioning

Under general anaesthesia, the patient is placed in a standard lateral decubitus position, with the abscess and lesion oriented upward and the lower limbs are flexed to relax the psoas muscle. The vertebral endplates should appear as a single line in both AP and lateral views, with the bilateral pedicle shadows equidistant from the spinous process in the AP view and completely overlapping in the lateral view.

2. Incision

Preoperatively, X-ray imaging is utilized to identify the location of the affected vertebral segment, and a mark is made on the left or right lower abdomen to indicate the involved intervertebral disc space and the positions of the surgical access points.

3. Surgical Procedure

**Fig. 1:**
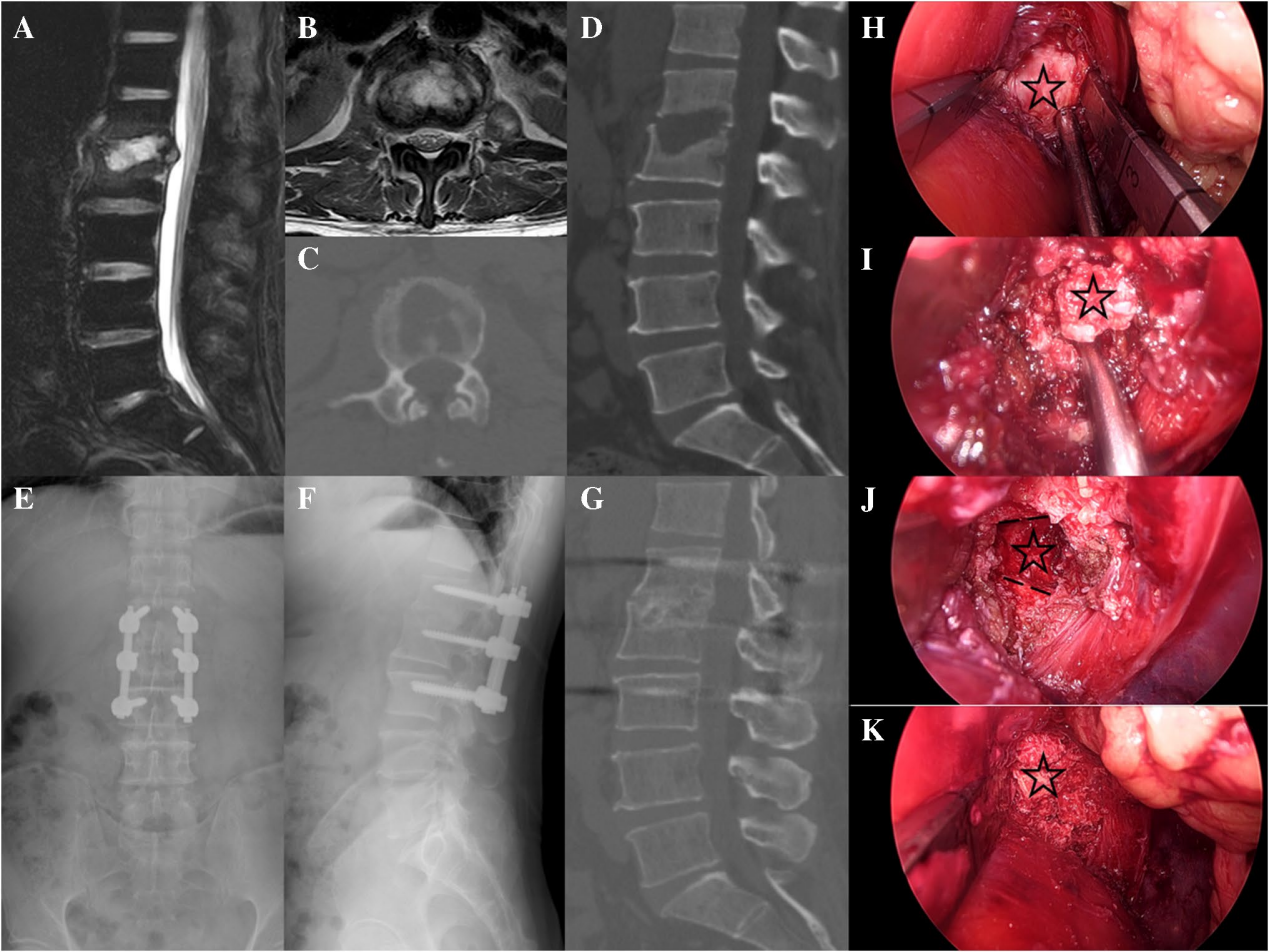
Case 3 **A**/**B**: Preoperative MRI demonstrates a lesion at the L1/2 level with an associated abscess causing spinal cord compression slightly. **C**/**D**: Preoperative CT reveals severe destruction of the L1/2 vertebral bodies. **E**/**F**:X-ray at 1 month postoperatively. **G**:CT at 12 months postoperatively confirmed complete fusion at L1/2. **H**: Exposure of the lesion, with the lesion marked by a black pentagram. **I**: Clearance of the lesion. **J**: Preparation of the bone graft bed, with the bone surface indicated by a black dashed line. **K**: Implantation of allogeneic bone

The surgical steps are as follows:a. a. An incision is made at the level of the affected intervertebral disc space, along the posterior wall of the intervertebral foramen. A customized balloon is employed to create a cavity with a volume of 500–600 mL. b. Trocar needles are inserted superiorly and inferiorly at the level of the affected intervertebral disc space. c. The iliopsoas muscle is dissected and exposed up to its anterior margin. d. At the intervertebral disc space, the iliopsoas muscle is retracted posteriorly to the posterior edge of the vertebral body. e. The intervertebral disc space and the margins of adjacent vertebral bodies are transversely exposed. f. The lesion is removed using a curette or osteotome. g. Following debridement, the surgical cavity is irrigated with normal saline. Bone grafting is performed to achieve fusion, a drainage tube is placed, and the incision is closed with sutures (Fig. 1).

4. All patients underwent posterior lumbar percutaneous pedicle screw fixation postoperatively.

### Outcome assessment

The operative time, operative blood loss, postoperative drainage volume, drain removal timing, preoperative and postoperative CRP/ESR follow-up results, postoperative complications, VAS scores, ODI scores, and the MNC were used to assess the effectiveness. The MNC evaluates clinical efficiency with "Excellent"(patient symptom-free, able to resume everyday life and work), "Good"(slight symptoms remain, activity slightly limited, no effect on life and work), "Fair"(some symptom relief, activity significantly limited, life and work affected) or "Pooг"(post-operation symptoms the same or worse as pre-operation symptoms).

### Statistical analysis

All the statistical analyses were conducted using SPSS version 23.0. Descriptive data are presented as the means and SDs. Student's t tests were used to compare continuous variables between two groups when the data were continuous, normally distributed, and homoscedastic. Chi-square tests and Fisher's exact tests were applied to evaluate differences between the two groups in other categorical variables. Differences during the postoperative follow-up period were analyzed by one-way variance analysis. p < 0.05 was considered to indicate statistical significance.

## Results

This study include 20 patients with LTB who underwent retroperitoneoscopic debridement, and their clinical data were analyzed. The infection levels ranged from L1/2 to L4/5, with four cases at L1/2(20%), four cases at L2/3(20%), nine cases at L3/4(45%), and three cases at L4/5(15%).

Comorbidities were present in 12 patients (60%), while eight patients (40%) had no associated medical illnesses. The most common comorbidities were: Hypertension (HTN): five cases (25%), Diabetes Mellitus (DM): five cases (25%), HTN and DM combined: three cases (15%). Among these patients, T-SPOT was positive in 18 (90%), and X-pert was positive in 19 (95%). All patients received a standard anti-TB regimen consisting of Isoniazid, Rifampicin, Pyrazinamide, and Ethambutol (HRZE) combined with Levofloxacin (V). Three patients (Cases 6, 11, and 16) also received Linezolid (Lzd). All patients exhibited a significant decrease in both CRP and ESR levels at the one month and three month postoperative assessments. (Tables 1 and 2).

Additionally, follow-up assessments demonstrated significant reductions in both VAS and ODI scores after surgery, which were closely associated with the debridement of the lesion. In contrast, no significant change in the kyphotic angle changes was observed, a finding likely attributable to the fixation. In this study, the kyphotic angle in the infective level was defined as the angle formed between lines drawn parallel to the superior endplate of the upper affected vertebra and the inferior endplate of the lower affected vertebra on sagittal reconstructed CT images. Interobserver reliability was assessed using Cohen’s kappa coefficient (κ) based on independent evaluations performed by two orthopaedic surgeons with more than ten years of experience before reaching consensus. The results demonstrated substantial agreement (κ = 0.78, 95% CI: 0.70–0.85) (Table 3).

This study also found that the mean operative time was 88.42 ± 7.07 min (range 70 ~ 105 min), mean operative blood loss was 26.32 ± 10.61 mL (range 15 ~ 40 mL) and mean postoperative drainage volume was 58.00 ± 11.31 mL (range 30 ~ 90 mL). All patients were followed for at least 12 months, the mean follow-up was 20.21 ± 1.41 months (range 18 ~ 24 months). Postoperative follow-up revealed that only one patient developed transient sensory abnormalities, which resolved after active treatment. No cases of infection recurrence or other serious surgery-related complications were observed in the remaining patients during the follow-up period (Table 4).

## Discussion

Tuberculosis is one of the oldest diseases in human history, and spinal tuberculosis (STB) is the most common form of extrapulmonary tuberculosis, with the lumbar spine being a frequently involved site, accounting for approximately 40% to 50% of spinal tuberculosis cases [11]. For patients with LTB without neurological or structural compromise, conservative treatment is the preferred and effective management strategy, with the majority achieving a cure through systematic non-operative approaches. However, with the delays in diagnosis, the failure rate of conservative treatment has been increasing, necessitating surgical intervention for patients who fail conservative management. Currently, there is significant controversy regarding surgical approaches for LTB [12]. We validate the efficacy and safety of retroperitoneoscopic debridement for the treatment of LTB.

In this study, the L3/4 level was the most frequently involved site, which is consistent with the anatomical predisposition of the lower lumbar spine to tuberculosis due to its high mobility and weight-bearing function [13]. Among the 20 patients, five had DM and five had HTN. The high prevalence of DM underscores its potential role as a risk factor for TB, likely attributable to impaired immune responses in diabetic patients [14, 15]. In contrast, no evidence was found to support an association between TB and HTN [16]. Notably, two cases exhibited discordant results between T-SPOT and Xpert tests, highlighting the complementary value of these assays in the diagnostic process [17].

**Fig. 2:**
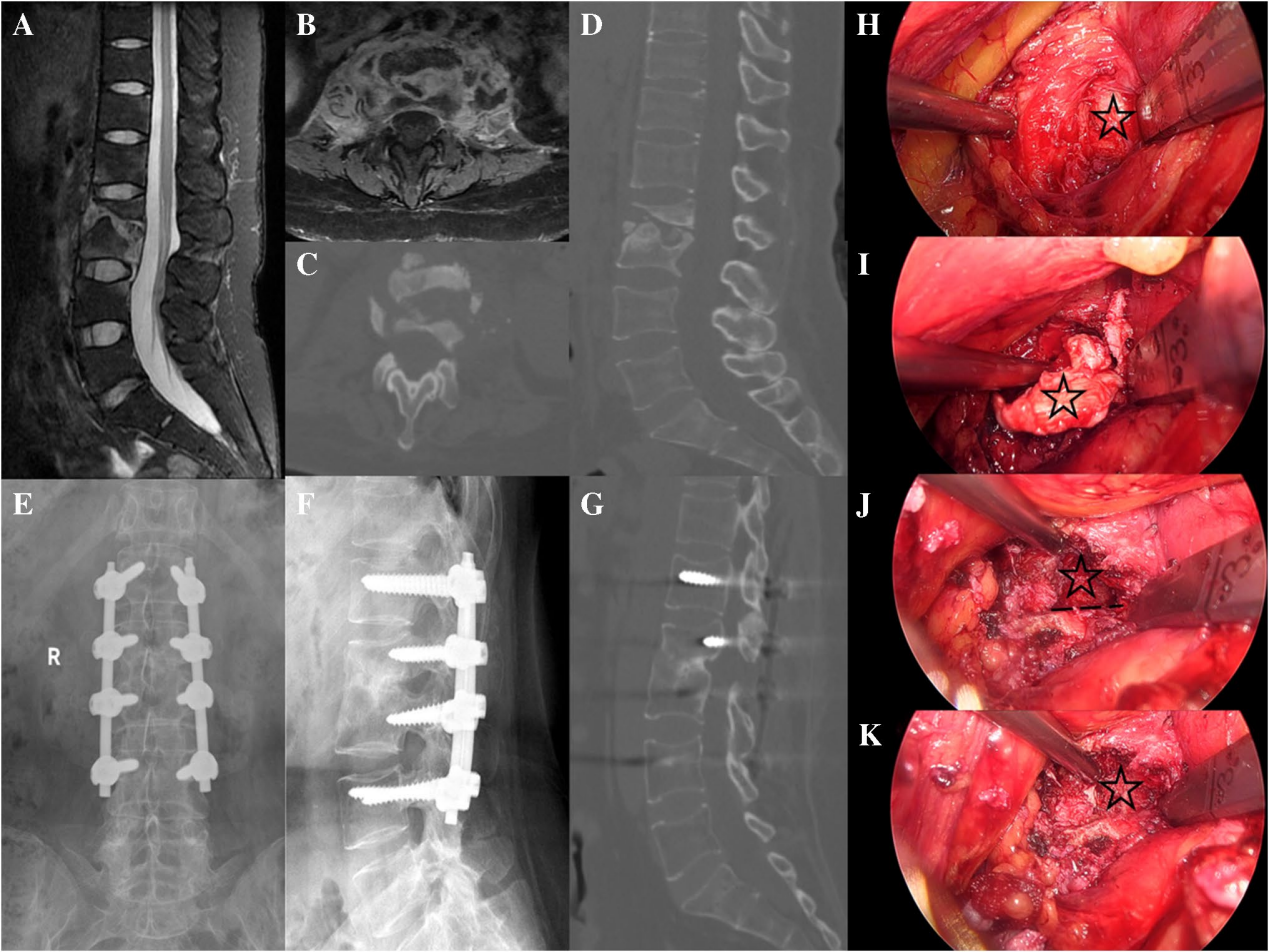
Case 6 **A**/**B**: PreoperativeMRI revealed abscess formation in the L2/3, with abscess formation both the psoas muscle. **C**/**D**: Preoperative CT demonstrated severe bone destruction in L2/3, with sequestrum formation in the L3 vertebra. **E**/**F**: X-ray at 1 month postoperatively. **G**:CT at 12 months postoperatively confirmed complete fusion at L2/3. **H**: Exposure of the lesion, with the lesion marked by a black pentagram. **I**: Clearance of the lesion. **J**: Preparation of the bone graft bed, with the bone surface indicated by a black dashed line. **K**: Implantation of allogeneic bone

**Fig. 3:**
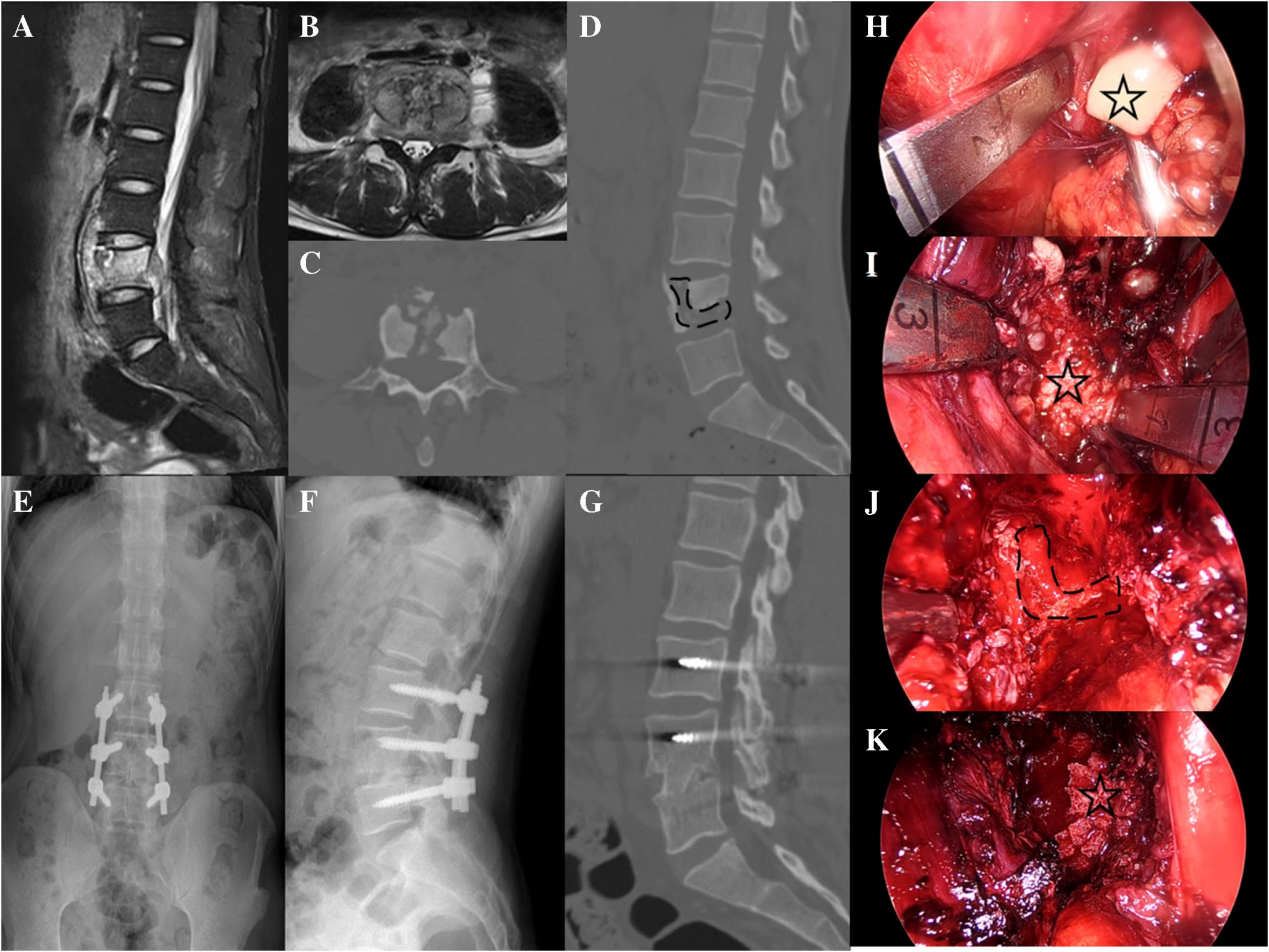
Case 10 **A**/**B**: Preoperative MRI revealed abscess both anterior and posterior to the L4, with abscess formation in the left psoas muscle. **C**/**D**: Preoperative CT demonstrated severe destruction of the L4, with notable destruction of the posterior vertebral wall .**E**/**F**:X-ray at 1 month postoperatively. **G**:CT at 12 months postoperatively confirmed complete fusion at L4/5. **H**: Drainage of purulent fluid, with the purulent fluid marked by a black pentagram. **I**: Exposure of the lesion, with the lesion marked by a black pentagram. **J**: Preparation of the bone graft bed, with the bone surface indicated by a black dashed line. **K**: Implantation of allogeneic bone

The principle of retroperitoneoscopic debridement lies in its utilization of a laparoscopic system to access lumbar lesions via the retroperitoneal space, thereby avoiding injury to abdominal organs. In 1996, Regan et al. [18] expanded the application of laparoscopy to the field of lumbar infectious spondylodiscitis, performing abscess drainage and interbody fusion via a transperitoneal approach. In the treatment of LTB, this approach enables direct exposure of the L1-L5 vertebral bodies, allowing for thorough abscess drainage, debridement of necrotic tissue, and interbody reconstruction, supplemented by local antibiotic irrigation and bone grafting [19]. Compared to open surgery, it minimizes muscle dissection and reduces the risk of peritoneal contamination, thereby lowering the incidence of postoperative infections and intestinal complications [20]. A retrospective study of 32 patients with LTB demonstrated that the retroperitoneoscopic debridement resulted in a mean operative time of 180 ± 45 min and an estimated blood loss of 150 ± 60 mL, which was significantly superior to the corresponding metrics of traditional open anterior approaches (*P* < 0.05) [21]. Additionally, compared with OLIF, Sun et al. [22] found that OLIF was associated with a higher complication rate, with cage subsidence being the most notable complication. Another study reported that the mean operative time for OLIF was 135.4 min and the mean blood loss was 107.1 ml, both of which were significantly higher than those observed in retroperitoneoscopic debridement [23]. Furthermore, this technique is crucial for preserving spinal stability. The simultaneous implantation of a titanium cage or autologous bone graft can effectively correct mild kyphosis caused by vertebral collapse, with postoperative radiographic follow-up showing a fusion rate of 96.43% (27/28) (Figs. 2 and 3). A multicenter case–control study further confirmed that for LTB patients with multi-level lesions, this procedure combined with posterior internal fixation enables single-stage treatment. A ten year follow-up demonstrated no progressive deformity, with a significant improvement in patients' quality of life [24]. A ten year follow-up demonstrated no progressive deformity, with a significant improvement in patients' quality of life [8]. This evidence indicates that retroperitoneoscopic debridement not only effectively eradicates the lesion but also facilitates early functional recovery, making it suitable for young and middle-aged patients and cases accompanied by mild-to-moderate deformities.

However, the advantages of this technique must be considered in comparison with other surgical approaches. Although the traditional posterior approach is technically straightforward, it provides inadequate exposure of anterior lesions, often necessitating a secondary procedure and resulting in increased cumulative trauma [25]. In contrast, the transperitoneal anterior approach offers excellent exposure but carries a significant risk of severe complications such as intra-abdominal adhesions and enteric fistula [26]. The retroperitoneal laparoscopic method skillfully avoids these pitfalls. A randomized controlled trial revealed a rate of major intraoperative vascular injury of only 1.2% with this technique, compared to 5.4% in the open anterior group (*P* < 0.05) [21]. Nonetheless, in cases with severe deformity or a high demand for multi-level fusion, this procedure may require supplemental posterior instrumentation to enhance biomechanical stability [27]. Overall, the retroperitoneoscopic debridement achieves a balance between minimally invasive access and comprehensive management, making it particularly suitable for LTB where imaging assessment indicates predominantly anterior pathology.

Despite its notable efficacy, retroperitoneoscopic debridement is associated with certain limitations and potential complications. Primarily, the procedure is technically demanding, requiring advanced laparoscopic skills and anatomical knowledge, which results in a steep learning curve and potentially prolonged operative times for novices. Secondly, CO₂ pneumoperitoneum may induce hypercapnia or pneumothorax, particularly in obese patients or those with a history of abdominal surgery. Clinical reports indicate an overall postoperative complication rate of approximately 8.5%, including transient low back pain (4.2%), nerve root irritation (2.1%), and superficial infection (2.2%) [10]. Although most are self-limiting, close monitoring is essential. Furthermore, exposure of the lower lumbar spine (L4-L5) can be somewhat limited, and manipulation near the bifurcation of the abdominal aorta and inferior vena cava carries increased risks. To mitigate these risks, meticulous preoperative CT/MRI evaluation and intraoperative neuromonitoring have become standard practice.

Looking forward, the application of retroperitoneoscopic debridement in LTB treatment will benefit from ongoing technological innovations. The introduction of robot-assisted systems and 3D-printed implants holds the potential to further enhance surgical precision and enable personalized spinal reconstruction [28]. Furthermore, within a multidisciplinary team (MDT) framework, integrating genotypic drug susceptibility testing for anti-tuberculosis regimens and AI-assisted radiographic diagnosis will optimize postoperative follow-up strategies. In conclusion, retroperitoneoscopic debridement has proven its value in clinical practice as a safe and effective minimally invasive intervention. However, its application requires careful consideration of individual patient factors to maximize therapeutic benefits.

This study has several notable limitations that temper the generalizability of its findings. First, it was conducted at a single institution with a relatively small sample size (*n* = 20), which limits statistical power and increases the risk of selection bias. Second, as a retrospective analysis without a concurrent control group, it lacks direct head-to-head comparison with other established approaches, such as open anterior debridement or posterior-only surgery. The definition and measurement of the kyphotic angle relied on independent assessments by two experienced orthopaedic surgeons, with substantial interobserver agreement (κ = 0.78). However, the absence of a standardized, blinded radiographic evaluation protocol represents a potential source of measurement variability. Furthermore, the inclusion criteria were deliberately restrictive which meaning the results may not apply to patients with multi-level involvement, severe kyphosis, or advanced neurological compromise. Long-term data beyond 12–24 months are also needed to fully evaluate fusion durability, deformity progression, and recurrence risk. Finally, while perioperative parameters were favorable, the technical demands of the procedure may restrict its reproducibility across centers with varying laparoscopic expertise.

## Conclusion

Retroperitoneoscopic debridement is an effective and reliable approach for treating LTB. This method efficiently clears the lesion, significantly improves patient symptoms, and is associated with low postoperative complication and infection recurrence.

## Data Availability

The datasets used and analysed during the current study are available from the corresponding author on reasonable request.

## Declarations

All authors listed meet the authorship criteria according to the latest guidelines of the International Committee of Medical Journal Editors. All authors are in agreement with the manuscript.
